# Social networks use in the context of Schizophrenia: a review of the literature

**DOI:** 10.3389/fpsyt.2024.1255073

**Published:** 2024-05-31

**Authors:** Carolina Suárez-Llevat, Beatriz Jiménez-Gómez, Carlos Ruiz-Núñez, Invención Fernández-Quijano, Eva María Rodriguez-González, Carlos de la Torre-Domingo, Iván Herrera-Peco

**Affiliations:** ^1^ Psychology Department, Faculty of Medicine, Universidad Alfonso X El Sabio, Madrid, Spain; ^2^ School for Doctoral Studies and Research in Biomedicine, Universidad Europea de Madrid, Faculty of Biomedical and Health Sciences, Madrid, Spain; ^3^ Department of Nursing, Human Nutrition and Dietetics, Universidad Europea de Madrid, Madrid, Spain; ^4^ Program in Biomedicine, Translational Research and New Health Technologies, School of Medicine, University of Malaga, Malaga, Spain; ^5^ Psychology Department, Faculty of Medicine, Alfonso X el Sabio University, Madrid, Spain; ^6^ Faculty of Health Sciences, Universidad Alfonso X el Sabio, Madrid, Spain

**Keywords:** disinformation, health literacy, mental health, schizophrenia, social networks

## Abstract

Schizophrenia is a persistent mental health condition that, while presenting challenges, underscores the dynamic nature of cognitive functions and encourages a unique perspective on how individuals engage with their surroundings. Social networks, as a means of communication of great importance at the present time, are for this type of people a way of interacting with their environment with a high level of security. The aim is to find out how schizophrenia is dealt with in different social networks and to differentiate between different types of articles dealing with the use of Facebook, X (former Twitter), YouTube, TikTok, Instagram, and Weibo. A total of 45 articles to i) Social networks used, ii) Country of analyzed users, iii) age of the users analyzed, iv) focus of the analyzed manuscript (mental health literacy, stigmatization, detection of patterns associated with schizophrenia, and Harmful substance use). It was observed that 45.45% of the studies analyzed were conducted in the USA population, followed by UK and China (13.64%). The most analyzed social networks were those based on audiovisual communication (60%). Furthermore, the two main foci addressed in these articles were: stigmatization of schizophrenia with 16 articles (35.55%), following by the prediction of schizophrenia-detecting patterns with 15 articles (33.33%) and the use of social networks to stigmatize people with schizophrenia (38%) and only 14 articles (31.11%) were focused on mental health literacy. Likewise, it was found that there is great potential in the use of the analysis of the content generated, as possible predictors of the presence of this disease, which would allow rapid detection and intervention for psychosis and schizophrenia.

## 1 Introduction

Nowadays, social media is considered a powerful tool to obtain information. This role allows them to be seen as a way for many citizens to obtain health-related information (1). So, easy access to social networks allows people to actively engage in the communication process and stay connected with each other (2). This makes a source of social influence, with the ability to help people express opinions on topics of interest, but to change attitudes and perceptions about situations (3) related to education or health among others (4, 5).

Schizophrenia is considered as a persistent mental health condition, characterized by shifts in perception, self-awareness, and behavior (6). People with schizophrenia may encounter challenges in social interactions or leading an independent life (7). However, it is crucial to note that interventions aimed at holistic recovery, which address cognitive and social aspects rather than just symptom reduction (7), enable people with schizophrenia to actively participate and integrate into social life.

As a result, people often face widespread social stigma related to their condition, which makes them hesitant to seek help from healthcare professionals for fear of being labeled as mentally ill (8, 9). In this context, the combination of social prejudice and the T anonymity offered by online platforms has led to the formation of support communities such as online community (10). An example of this has been described during the COVID-19 pandemic, and the ensuing lockdown, where some patients perceived the lockdown positively, as it improved their relationship with staff, fostered a greater sense of community and emphasized self-awareness and treatment (11). However, pandemic-induced stress and negative affect, as well as isolation, were associated with an increased risk of psychotic symptoms (12).

It is important to note that social networks can play a critical role as a resource for health-related information when needed (13, 14). Given this potential, it is important to take advantage of social networks to identify these individuals and offer them support (15, 16).

Nowadays some social networks are playing an increasingly important role in the search for health information (14, 17), especially among young (18) and young adults (18-24 years of age), who use them very frequently and spend considerable time in platforms such as Instagram, YouTube, Twitter or TikTok (18–20).

However, the main drawback commonly described for health information disseminated through social networks is the lack of quality and reliability (20, 21). Individuals may be vulnerable to unreliable content and may not have adequate tools to successfully filter reliable information from untrue content (4). In terms of mental health, another perspective should also be considered: individuals’ exposure on social networks can lead to social contagion, or peer pressure, to conform to certain behaviors or actions (21–23). Not to mention that some statements on social networks may promote rejection towards people with mental illnesses, as may be the case with people diagnosed with schizophrenia (23, 24).

Beyond the negative effects associated with information disseminated through social networks, we should highlight the potential use of these platforms as channels to promote mental health care for people with disorders such as schizophrenia. Social networks, serves as a way of communication for people, through participation in online networking that helps provide emotional support and a sense of belonging, as well as the opportunity to share experiences with people facing similar challenges (16). Social networks, also serve as outlets to provide reliable information generated by mental health professionals (14, 24), or to disseminate information to raise awareness and reduce the stigma associated with mental illness (14, 25).

To the best of the researchers’ knowledge, there is no previous review that has analyzed how users of different social networks, whether text-based, such as Twitter or Weibo, or audio-visual, such as Youtube, TikTok or Instagram, deal with schizophrenia. Whether they are people who suffer from it, family members or other users. We believe that this review will help to better understand how schizophrenia is addressed in different social networks.

The present research was three main aims, were: i) analysis the existing international literature on schizophrenia and social networks, ii) to analyze how schizophrenia is handled by users from different social networks. assessing information on the countries where the studies took place, the age of the people included in the studies, or whether these articles were associated with stigmatization or other approaches to schizophrenia, and iii) Comparing whether the COVID-19 lockdown is reflected in any way in the documents published during that period.

## 2 Materials and methods

### 2.1 Search methodology

To identify studies of interest, a bibliographic search was conducted covering a wide range of published health-related research from the following databases: Medline, Web of Science and Scopus. The time frame of the study included articles published from January 1, 2017, to December 13, 2022.

The search terms used in the different databases were focused on obtaining the appropriate result to answer the objectives set out in this study, these are as follows: terms used for schizophrenia included “schizophrenia*” OR “psychosis”, On the other hand, search terms related to social networks included: “social networks” OR “Facebook” OR “Twitter” OR “Instagram” OR “Weibo” OR “TikTok” OR “YouTube”. In relation to social networks, and more specifically about Twitter. At the time of the search this social network kept this name since the denomination of X was given by Elon Musk in July 2023.

An example of the search syntax used in SCOPUS search engine was as follows: (schizophrenia OR psychotic disorders OR psychotic) AND (social networks OR Facebook OR Twitter OR Instagram OR Weibo OR TikTok OR Youtube)

The following criteria were defined to retrieve eligible studies for this review. Inclusion criteria were defined as: i) articles written in English, ii) articles published in a peer-reviewed journal, iii) articles that clearly stated the methodology and results, iv) articles in which the researchers used Facebook, Twitter, Instagram, Weibo, TikTok or YouTube to obtain results, v) articles published the last 6 years, between January 1, 2017 and December 31, 2022. One of the aims of the present study was to observe whether the blockade triggered by the COVID-19 pandemic had any effect on the use of social networks associated with schizophrenia. To this end, given that the blockade was declared in many countries in the year 2020, from January to April (26), we aimed to approach the study from 3 years before the first blockade to 3 years after.

Exclusion criteria were defined as i) non-research articles (editorials, commentaries, preprints, abstracts, proceedings, book reviews, reviews, etc.), ii) articles focusing on psychiatric disorders other than schizophrenia, iii) articles focusing on social networks not included in the inclusion criteria.

### 2.2 Data collection

After retrieving the studies from the databases, duplicate reports were removed, and the titles and abstracts of the remaining articles were screened to exclude studies that did not meet the eligibility criteria. To avoid error and bias, three independent researchers conducted the review process to identify articles that met the inclusion criteria, using the Zotero bibliographic reference manager, which allows for the detection and elimination of duplicate articles (27). Titles and abstracts were then analyzed to exclude irrelevant articles. Finally, the full texts were evaluated using PRISMA criteria (28), to determine whether the articles met the eligibility criteria. During this selection phase, any disagreements among the investigators were resolved by discussion and consultation with a reviewer who was not actively involved in the study selection. The quality assessment for each studied was assessed using the tool of the National Heart, Lung, and Blood Institute (NIH) (https://www.nhlbi.nih.gov/health-topics/study-quality-assessment-tools) for cross-sectional studies that included 14 criteria (29). Two independent researchers assessed each article. We rated the studies as ‘Good’ if the count was ≥11, ‘Fair’ if the count was between 5 and 10, and ‘Poor’ if the count was ≤4. The studies generally had a fair quality (moderate risk of bias). The reliability of this analysis was evaluated using an analysis to assess the correlation between valuations, the Cohen’s kappa coefficient (0.72), which implies a good correlation between the valuations.

### 2.3 Data analysis

After selecting the relevant articles, the data were compiled in a Microsoft Excel spreadsheet. Due to the heterogeneity of the studies included in the present analysis, a narrative synthesis was performed according to context analysis associated with schizophrenia. We grouped the manuscript into the following categories: i) Social networks, where the type of social network used in the studies was detailed. ii)Nationality of users analyzed in the manuscripts. iii) Age of the users analyzed in the manuscript. iv) Main focus of the analyzed manuscript,

Within the fourth category, the focus revised were: i) Mental Health Literacy, focusing on the use of social networks as, use for schizophrenia education, ii) Prediction/detection of patterns associated with schizophrenia, iii) Addictions. iv) Stigmatization & Trivialization of schizophrenia, Stigmatization, is understood as the exposure of how social networks can be used to disseminate biased information about schizophrenia and negatively target schizophrenia patients. Trivialization is understood as considering the disease as unimportant and downplaying its implications for those who suffer from it.

Finally, for data analysis, descriptive and inferential statistics were used via the Jamovi (Version 2.3.11, The Jamovi Project, Sydney, Australia). The categorical variables included in the present study and derived from the subcategories defined in the qualitative approach, have been expressed as the total number of individuals and proportions. Furthermore, the comparison between groups was performed with a non-parametric test, the chi-square test, since this test does not require homoscedasticity in the data and permits the evaluation of dichotomous independent variables. The statistical level of significance was set at p < 0.05.

## 3 Results

### 3.1 Selection of evidence sources

**Figure 1:**
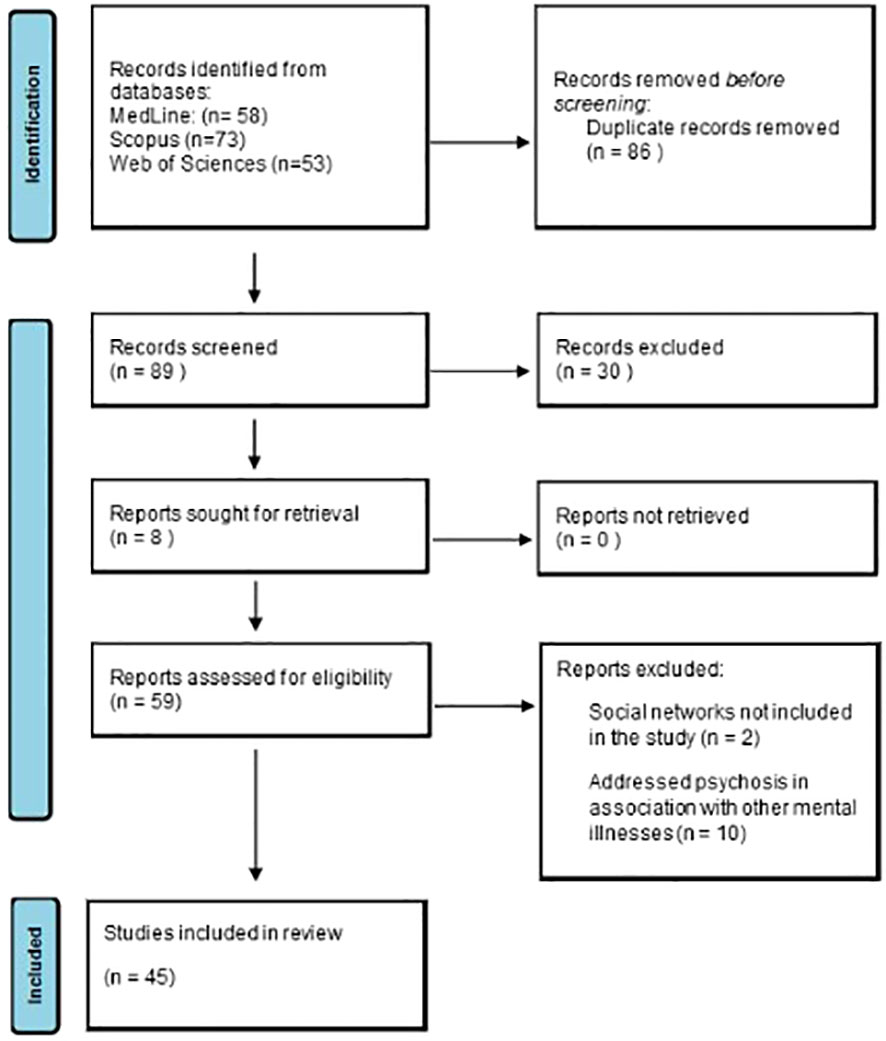
PRISMA flowchart outlining the review process.

The search strategy yielded 184 articles. After eliminating duplicates, a total of 89 articles were screened for eligibility criteria (**Figure 1**). Of these, 44 articles were eliminated due to lack of assessment, leaving 45 articles for full-text analysis (**Table 1**).

**Table 1:** Social networks and Schizophrenia.

Authors; Year and reference	Country	Age	Social networks analyzed	Social networks analyzed (SNA), and focus of the manuscript (F)	Category
Battaglia, AM, et al., 2022 (24)	Not specified	Not specified	Instagram	SNA: Instagram F: Use of images and videos with negative information about schizophrenia.	Stigmatization of schizophrenia
Fonseka, LN &Woo, BKP. 2022 (14)	Not specified	Not specified	Facebook, Twitter, Instagram, YouTube	SNA: Facebook, Twitter, Instagram, YouTube. F: implementation of machine learning, through text analysis. But also, analysis of colors and faces in the images.	Prediction/detection of patterns
Franco, OH, et al., 2022) (30)	USA	18-32 years old	Facebook, Twitter	SNA: Facebook, Twitter. F: The frequency of posting messages was analyzed. A higher frequency was associated with greater severity of symptoms.	Prediction/detection of patterns
Birnbaum, ML, et al., 2017 (31)	Not specified	Not specified	Twitter	SNA: Twitter. F: A text analysis of self-reported schizophrenia users was conducted. They were evaluated by mental health professionals to confirm this assumption. Patterns of diagnosis identification were sought.	Prediction/detection of patterns
Birnbaum, ML, et al., 2019 (32)	USA	15-35 years old	Facebook	SNA: Facebook. F: Machine learning techniques were used, using posts from patients who were admitted to hospitals for a psychotic break. The object is to detect patterns that can help detect people who may be close to suffering a psychotic break.	Prediction/detection of patterns
Ashok, N, et al., 2021 (33)	Not specified	Not specified	Twitter	SNA: Twitter. F. Machine learning techniques are used, to detect patterns associated with schizophrenia in Twitter message text.	Stigmatization of schizophrenia,
Kelly, DL, et al., 2020 (34)	USA	18-64 years old	Facebook	SNA: Facebook. F: Assessment by clinical experts. Both of Facebook posts, versus in-person interview assessment. Correlation assessment between diagnoses	Prediction/detection of patterns
Wong, KTG, et al., 2020 (35)	Australia	18-64 years old	Facebook	SNA: Facebook. F. Evaluation of the use of the Internet and the confidence of patients with schizophrenia in the information they obtain from this medium. It notes that these patients use it little and would be unsuitable for interventions or for educational action.	Mental Health Literacy
Birnbaum, M.L., et al. (2020) (36)	USA	15-35 years old	Facebook	SNA: Facebook. F: Analyzing text and images uploaded to your posts. In the texts there is a predominance of words associated with perception. A higher frequency of posts is observed at times when they follow a hospitalization due to an outbreak.	Prediction/detection of patterns
Fekih-Romdhane, F., et al. (2022) (37)	Not specified	Not specified	Facebook	SNA: Facebook. F: Analysis of the risk of falling into technology addiction in the case of people with diagnosed schizophrenia.	Schizophrenia and adictions
Hswen, Y., et al. (2017) (38)	Not specified	Not specified	Twitter	SNA: Twitter. F: Analysis of the relationship between schizophrenia and addictive habits such as smoking. The study assessed the frequency, timing, and type of communication about tobacco-related content on Twitter among users who self-identified as schizophrenic. Those users showed significantly higher odds of tweeting about tobacco.	Schizophrenia and adictions
Lustig, A., et al. (2021) (39)	Not specified	Not specified	YouTube	SNA: YouTube. F: This study focuses on the analysis of information about schizophrenia. Specifically, to gangstalking which is a novel belief system where patients believe they are persecuted, harassed by complicit perpetrators and deny mental illness.	Mental Health Literacy
Passerello. G.; et al. (2019) (40)	Not specified	Not specified	Twitter	SNA: Twitter. F: Assessment of attitudes towards schizophrenia and psychosis. The terms psychosis/psychotics are associated with significantly more tweets with negative content than schizophrenia/schizophrenics.	Stigmatization of schizophrenia,
Lam, N.H.T.& Woo, B.K.P. (2020) (41)	China	18-34 years old	Instagram, Facebook, YouTube	SNA: Instagram, Facebook, YouTube. F: The use of different social networks for the dissemination of information about schizophrenia is evaluated. From texts on Facebook, videos on YouTube as well as the evaluation of the effectiveness of the use of Instagram in psychoeducation on first episodes of psychosis and schizophrenia.	Mental Health Literacy
Alvarez-Mon, M.A., et al. (2021) (42)	Not specified	Not specified	Twitter	SNA: Twitter. F: Study analyzing the use of antipsychotic medication in schizophrenic patients, being the patients who develop the conversation. Trivialization of the use of some medication is observed.	Stigmatization of schizophrenia,
Jilka, S., et al. (2022) (43)	Not specified	Not specified	Twitter	SNA: Twitter. F: Analysis of texts posted on Twitter, focused on analyzing the contents of stigmatizing Tweets. They use Machine learning to learn patterns to recognize stigmatizing content.	Stigmatization of schizophrenia,
Alvarez-Mon, M.A., et al. (2019) (44)	Not specified	Not specified	Twitter	SNA: Twitter. F: Application of an algorithm to Tweets, specifically focusing on hashtags associated with schizophrenia. It is observed that there is a high dissemination of inaccurate information in tweets about psychosis and schizophrenia, leading to the stigmatization of the illness	Stigmatization of schizophrenia,
Li, A., et al. (2020) (45)	China	Not specified	Weibo	SNA: Weibo. F: A review of word frequency is conducted utilizing the Waikato Environment for Knowledge Analysis software to categorize messages stigmatizing schizophrenia. The algorithm is also trained to differentiate stigmas associated with schizophrenia from those associated with depression.	Stigmatization of schizophrenia,
Kara, U.Y. & Şenel, K.B. (2022) (46)	Turkey	Not specified	Twitter	SNA: Twitter. F: Analysis of tweet contents revealed a high frequency of messages stigmatizing individuals with schizophrenia	Stigmatization of schizophrenia,
SariogluKayi, E., et al. (2017) (47)	Not specified	Not specified	Twitter	SNA: Twitter. F: Study focused on the analysis of tweets from individuals indicating they suffer from schizophrenia. Analysis of semantic, syntactic, and pragmatic patterns	Prediction/detection of patterns
Delanys, S., et al. (2022) (48)	Not specified	Not specified	Twitter	SNA: Twitter. F: Analysis of a conversation about schizophrenia. The appropriate use of medical terms in relation to schizophrenia was reviewed. It was observed that there is significant confusion in the terms, leading users to be unclear in expressing their contribution	Mental Health Literacy
Jansli, S.M., et al. (2022) (49)	United Kingdom	>18 years old	Twitter	SNA: Twitter. F: Study focused on mental health professionals, querying them about their Twitter activity during the COVID-19 pandemic and its impact on the stigmatization of diseases such as schizophrenia	Stigmatization of schizophrenia,
Robinson, P., et al. (2019) (50)	Not specified	Not specified	Twitter	SNA: Twitter. F: The trivialization or stigmatization of schizophrenia was analyzed. This categorization was performed by two of the authors of the document. Subsequently, an analysis of the frequency of term occurrences was conducted	Stigmatization of schizophrenia,
Naslund, J.A., et al. (2019) (51)	USA, Canada, United Kingdom, among others	All ages	Twitter	SNA: Twitter. F: The participants were presented with the danger of using social media, with the main finding being the existence of fear of being judged and stigmatized. However, it was also observed that being identified as schizophrenic could potentially disrupt their ability to interact with others	Stigmatization of schizophrenia,
Bowen, M., & Lovell, A. (2021) (52)	United Kingdom	Not specified	Twitter	SNA: Twitter. F: Articles focused on an analysis of the content of Tweets sent by the British press regarding schizophrenia. It is found that negative news about schizophrenia predominates, promoting stigma and misinformation among the population	Stigmatization of schizophrenia,
Ernala, S.K., et al. (2018) (53)	Not specified	Not specified	Twitter	SNA: Twitter. F: This study discusses the implications of schizophrenia disclosures on Twitter, as well as its stigmatization. It emphasizes the creation of socially appealing and supportive online spaces for the disclosure of stigmatized mental illnesses	Stigmatization of schizophrenia,
Abdel-Baki, A., et al. (2017) (54)	United States	18-32 years old	Facebook, Twitter	SNA: Facebook, Twitter. F: Study on the use and access of technology for youth experiencing a first psychotic episode, including gaming activities, to inform the future development of therapeutic applications for education and management of the schizophrenia.	Mental Health Literacy
Bhoi, D. &Thakkar, A. (2020) (55)	Not specified	Not specified	Twitter	SNA: Twitter. F: Sentiment analysis to enhance the lives of patients with schizophrenia. Various tools and methodologies are employed, from which color-coded word clouds can be generated based on sentiment. This approach aims to recognize and understand the feelings of patients in the future	Prediction/detection of patterns
ColderCarras, M., et al. (2018) (56)	Not specified	Not specified	Twitter	SNA: Twitter. F: The study provides information on the use of social networks, by a population of English-speaking community psychiatry patients, who attended an outpatient program over a period of 4 weeks	Mental Health Literacy
Sangeorzan, I., et al. (2019) (57)	Not specified	Not specified	YouTube	SNA: YouTube. F: This study analyzes the experiences of individuals with self-identified serious mental illnesses who vlog about their condition on YouTube. Observing negative or stigmatizing experiences	Stigmatization of schizophrenia,
Merchant, R.M., et al. (2019) (58)	United States	Not specified	Facebook	SNA: Facebook. F: A study that links electronic health records with social media data from consenting patients, identifying that patients’ Facebook status updates can predict various health conditions. This suggests opportunities to use social media data to determine the onset or exacerbation of diseases and to conduct social media-based health interventions	Prediction/detection of patterns
Hswen, Y., et al. (2018) (59)	Not specified	Not specified	Twitter	SNA: Twitter. F: The study examined the feasibility of monitoring online discussions about suicide among Twitter users who identify themselves as schizophrenic. Twitter users self-identifying as schizophrenic posted more tweets about suicide	Prediction/detection of patterns
Hernandez, M.Y., et al. (2020) (60)	Not specified	Not specified	Twitter	SNA: Twitter. F: This study explored trends in health information exchanged by patients, doctors, and health organizations regarding schizophrenia through the analysis of tweets using #schizophrenia. Most tweets focused on improving schizophrenia literacy, followed by personal experiences/motivational stories and biological explanations of the disorder. Logistic regression results indicated that, compared to doctors, patients were less likely to tweet with an academic source	Mental Health Literacy
Rekhi, G., et al. (2019) (61)	Singapore	21-65 years old	Facebook, Twitter, Instagram, YouTube	SNA: Facebook, Twitter, Instagram, YouTube. F: Study focused on the prevalence of social network use and its association with symptoms in individuals with schizophrenia. Frequency of use and types of posts were collected and both positive (PANSS scale) and negative (CAINS scale) symptoms were assessed. The use of social networks was associated with the presence of negative and depressive factors as well as motivation-pleasure. It is suggested that assessing the use of social networks should be included in the clinical approach to patients.	Prediction/detection of patterns
Saha, K., et al. (2017) (62)	United States	>13 years old	Facebook	SNA: Facebook. F: this study constructs an index that measures the knowledge of different demographic groups about schizophrenia-related information on Facebook; study how this index differs between demographic groups and how it correlated with complementary Web-based (Google Trends) and non-Web-based variables on population well-being (mental health and infrastructure indicators); and examine the relationship of the Facebook-derived schizophrenia index to other types of online activity, as well as offline health and mental health outcomes and indicators.	Prediction/detection of patterns
Lampropoulos, D., et al. (2022) (63)	France	Not specified	Facebook	SNA: Facebook. F: Study focused on the analysis of schizophrenia activism on French-speaking pages on Facebook. Assessment of two activist pages presenting differences in their organization (e.g., funded or not) and how they work for the training and social interaction of people with schizophrenia.	Mental Health Literacy
Retnowati, Y. (2017) (64)	Indonesia	Not specified	Facebook	SNA: Facebook. F: Study analyzing the educational strategy of Indonesian Community Care for Schizophrenia (ICCS) in providing its psychoeducation services on Facebook and face-to-face. A sample of people with schizophrenia and their families in a caregiver role were surveyed for their interpretation of information ICCS posts on Facebook	Mental Health Literacy
Lam, N.H.T., et al. (2017) (65)	USA	>34 years old	YouTube	SNA: YouTube. F: This study explores the role of YouTube in delivering schizophrenia education to people in the U.S. who are also fluent in Chinese. Three psychoeducational videos related to schizophrenia were uploaded to YouTube. Data were collected over a 12-month period and assessed both views and comments interactions, with video	Stigmatization of schizophrenia,
Kumar, D. &Jha, M. (2018) (66)	Not specified	Not specified	Youtube	SNA: YouTube. F: Evaluation of the quality and reliability of YouTube videos on psychosocial interventions for people with schizophrenia. Most of these videos have been posted by professionals or professional groups that present information in a simple manner and with reliable content.	Prediction/detection of patterns
Nour, M.M., et al. (2017) (67)	Not specified	Not specified	YouTube	SNA: YouTube. F: This study analyzes the accuracy of depictions of psychosis in the context of a diagnosis of schizophrenia (referred to in this article as “acute schizophrenia”) on YouTube and evaluate the usefulness of these videos as educational tools for medical education and to help students recognize the clinical features of acute schizophrenia.	Mental Health Literacy
Godwin, H.T., et al. (2017) (68)	Not specified	Not specified	YouTube	SNA: YouTube. F: Analysis of the educational potential and effectiveness of a 3-minute video clip of a schizophrenia simulation posted online on YouTube. Authors examined the 267 public comments posted on the video-sharing website YouTube over 8 years by viewers of a schizophrenia simulation video titled “virtual hallucinations” made on the gaming platform Second Life. The comments were independently categorized into six groups, then cooperatively finalized, and qualitatively analyzed.	Mental Health Literacy
Aguiar, J.P., et al. (2022) (69)	Not specified	Average age 35 years old	Facebook, Twitter	SNA: Facebook, Twitter. F: Study focused on the knowledge and assessment of health professionals’ knowledge of pharmacotherapy in the elderly. Potentially Inappropriate Drugs that may increase the likelihood of adverse events, especially in elderly patients with mental health disorders, such as schizophrenia.	Mental Health Literacy
Saha, K., et al. (2019) (70)	Not specified	Not specified	Twitter	SNA: Twitter. F: Development of machine learning models to assess effects related to mood, cognition, depression, anxiety, psychosis, and suicidal ideation. Then, based on a causal analysis based on a stratified propensity score, we observe that specific drug use is associated with characteristic changes in an individual’s psychopathology. The model is proposed to serve as a predictor of treatment outcomes.	Prediction/detection of patterns
Woo, B.K.P. & Kung, E. (2018) (71)	China	Not specified	YouTube	SNA: YouTube. F: YouTube video intervention to promote education about the first episode of psychosis in Chinese people as they report that the quality of mental health is lower in Chinese than in English. Interactions with youtube videos are valued.	Mental Health Literacy
Hansen, H., et al. (2019) (72)	Norway	Not specified	Facebook	SNA: Facebook. F: Narrative discourse analysis of an information campaign aimed at help-seeking in the first episode of psychosis. A large sample of information material used by TIPS Stavanger University Hospital (Norway) was examined. The material consisted of posters, booklets and brochures, newspaper ads, Facebook ads, and the TIPS Info website, representing various campaigns from 1996 to April 2018. A discursive narrative approach was applied at the epistemological level. At the practical level, a team-based thematic analysis was used to identify patterns in the data.	Mental Health Literacy

Of the articles removed, the 29 that were initially withdrawn were because they met one of the exclusion criteria, such as being a book chapter. The remaining 15 articles were removed after an in-depth review when it was found that the articles did not focus on schizophrenia but on psychosis, without specifying their association with schizophrenia or dealing with social networks other than those asked, such as reddit, or using mobile phone messaging applications.

### 3.2 Social networks related to schizophrenia

When analyzing the social networks in this study, it was found that the most frequent social network was Twitter, appearing in 54.17% of the documents (n=26). It was followed by Facebook, present in 37.5% (n=18) of the documents. Youtube appeared in 20.83% (n=10), followed by Instagram which appeared in 8.33% of the documents (n=4).

Finally, Weibo, another text-only social network exclusively accessible in China, appeared in 2.08% of the documents (n=1). It is noteworthy that the social network Tiktok, at the time of this review, will not have any documents.

Furthermore, when the number of social networks was analyzed, we observed that several studies, 87.5% (n=42) focused on only one social network, while the 12.5% (n=6) focused on two or more social networks (**Table 1**). Finally, in relation to the typology of social networks, it was found that 77.08% (n=37) of the documents analyzed social networks focused on the use of text. This compares to 16.67% (n=8) that analyzed audiovisual social networks. The remaining 6.25% (n=3) analyzed social networks of both types (**Table 1**).

It is striking that TikTok, the social network currently booming worldwide, is not being used to share information related to schizophrenia. The most relevant platforms for this topic are Twitter, Instagram or YouTube, platforms that focus on sharing videos or audiovisual content. Therefore, we can say that this is a central aspect when it comes to disseminating information to the population.

### 3.3 Geolocation and age of studies, about schizophrenia, included

Regarding the geographical location of the studies, it was found that 56% (n=28) did not provide this category of information, 40% (n=20 was targeted at users coming from a single country, and, finally, the 4% (n=2) analyzed users from different countries.

**Figure 2:**
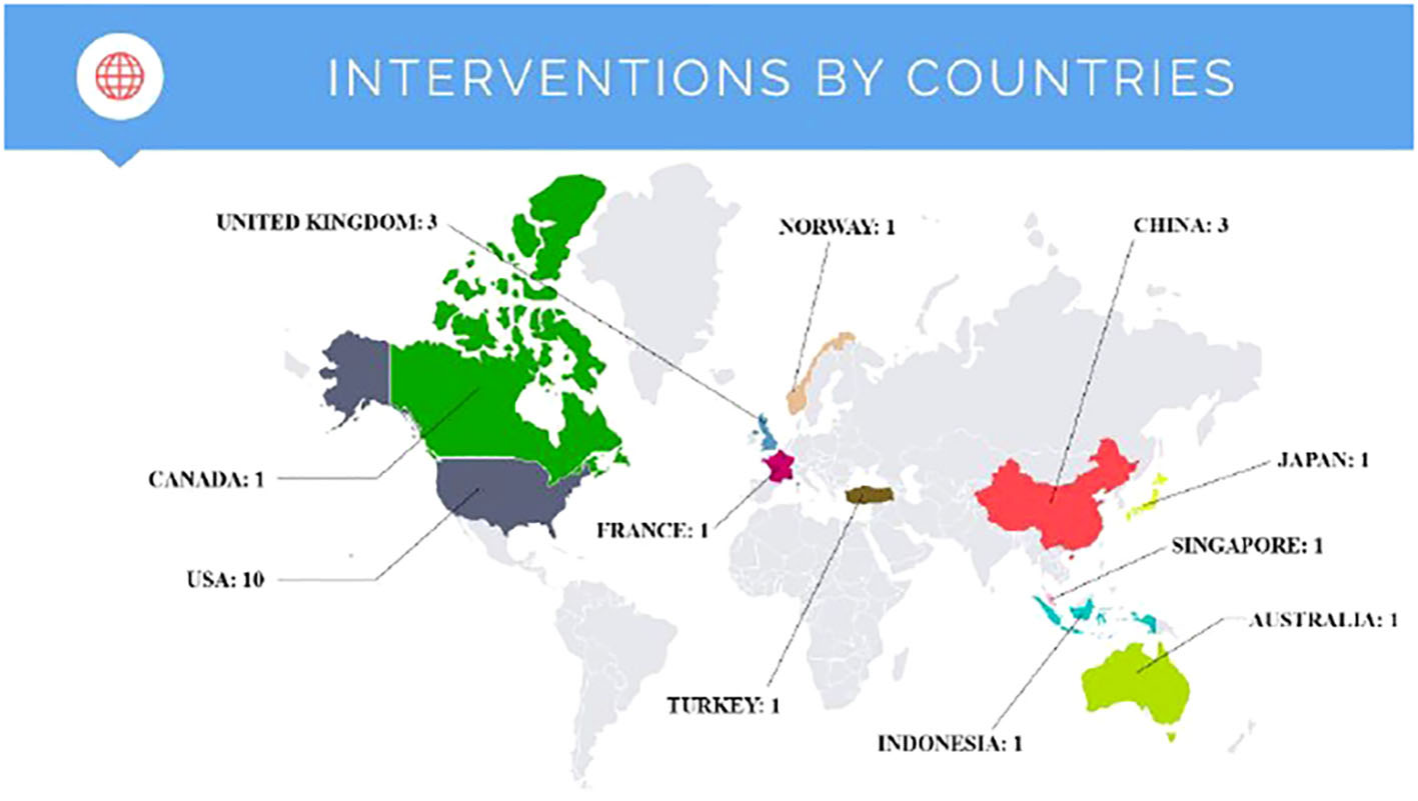
Description of the countries on which the analyzed studies focus.

Of the 22 countries that provided the countries of origin of the users analyzed, the country that appeared most frequently was the USA, which appeared in 45.45% (n=10) of the manuscripts. This was followed by China (n=3) and United Kingdom (n=3) with 13.64%. Finally, Australia, Canada, France, Indonesia, Japan, Norway, Singapore, and Turkey appeared in only one study (see **Figure 2**).

About age, only 13 (27.08%) of the 48 studies included provided information on the age of the users analyzed. However, it is important to note that the age range of users was observed to be very disparate between the different studies, making comparison between studies complexes. It is worth noting that, about the adolescent population, there are 4 studies that indicate that they analyzed populations aged 13, 15, and 16 years and older. Thus, of those studies that do analyze age, only 4 studies (30.77% of total studies that provide the participants’ age) indicated that they analyzed users who indicated an age of less than 18 years, but in no case did they focus exclusively on children or adolescents (**Table 1**). Likewise, no study defining age indicated that groups of people aged 65 years or older were analyzed. Finally, 6 studies (46.15%) were found to focus on the group defined as adults aged no more than 35 years. 1 study (7.14%) focused on adults over 34 years old (**Table 1**).

### 3.4 Approaches to schizophrenia conversation on social networks

The results are developed below according to the previously defined categories.

When analyzing the category of Mental Health literacy, it was observed that a total of 16 documents focused on addressing the use of social networks (**Table 2**). It should be noted that 13 social networks that used text were analyzed, compared to 6 articles that used social networks based on audiovisual communication (**Table 2**).

**Table 2:** Social networks associated with the focus of the manuscripts included in the systematic review.

						Freq. Ap. by categories (% total)			
				Total	Freq. Ap by social networks (% total)	MHL	PoP	S&A	Stig.
**Social Networks**	**Twitter**	**Global**		25		5 (20%)	8 (32%)	1 (4%)	11 (44%)
		**Lockdown**	BL	13	52%	2 (15.39%)	5 (38.46%)	1 (7.69%)	5 (38.46%)
			AL	12	48%	3 (25%)	3 (25%)		6 (50%)
	**Facebook**	**Global**		16		7 (43.75%)	8 (50%)	1 (6.25%)	0
		**Lockdown**	BL	7	43.75%	3 (42.86%)	4 (57.14%)	0	0
			AL	9	56.25%	4 (44.44%)	4 (44.44%)	1(11.12%)	0
	**Weibo**	**Global**		1	0	0	0	0	1
		**Lockdown**	BL	0	0	0	0	0	0
			AL	1	100%	0	0	0	1 (100%)
	**Instagram**	**Global**		4	0	1 (25%)	2 (50%)	0	1 (25%)
		**Lockdown**	BL	1	25%	0	1	0	0
			AL	3	75%	1 (33.33%)	1 (33.33%)	0	1 (33.33%)
	**TikTok**	**Global**		0	0	0	0	0	0
		**Lockdown**	BL	0	0	0	0	0	0
			AL	0	0	0	0	0	0
	**Youtube**	**Global**		10		5 (50%)	3 (30%)	0	2 (20%)
		**Lockdown**	BL	7	70%	3 (42.86%)	2 (28.57%)	0	2 (28.57%)
			AL	3	30%	2 (66.66%)	1 (33.34%)	0	0

Where: Freq. Ap. Means Frequency of appearance. MHL, means Mental Health Literacy, PoP means Prediction/detection of patterns. S&A, means Schizophrenia and addictions. Stig., means Stigmatization. In relation with the COVID-19 lockdown, BL, means Before the Lockdown, and AL, means After the Lockdown.

It is found that authors such as Wong, KTG, et al. (30) who are based on a text-based social network such as Facebook, indicate that people with schizophrenia make little use of social networks, making it difficult to use them to develop a mental health education intervention. This data contrasts with other studies, which do indicate that the use and presence of social networks supports mental health education (52, 54, 58–60, 66, 69).

It Is worth highlighting the study by Delanys, S., et al. (43) in the study they carried out on Twitter, which defined that training on what and how to use medical terms appropriately was the responsibility of healthcare professionals.

Finally, the work of Retnowat, Y (61)., focuses on the analysis of the communication and training strategy on mental health focused on schizophrenia, on Facebook.

When assessing the use of audiovisual social networks and their use to develop training activities in mental health, two documents were found that support psychoeducational intervention on the social network Youtube (34, 62, 65, 68). A Likewise, in relation to YouTube, Nour, M.M., et al. (64) analyze the importance of YouTube as a tool for mental health training for future medical professionals. Finally, Lam, N.H.T. & Woo, B.K.P. (36), demonstrated that the use of psychoeducation advertisements on Instagram had the same or better results than training on Facebook. A total of 15 articles were found to focus on identifying patterns that would allow them to detect schizophrenia through social network messages (**Table 1**).

Within these documents, could be identified those that focused on the development of machine learning as an analysis strategy to detect patterns that facilitate the identification of people with possible behaviors associated with schizophrenia (27, 28, 37, 38, 53). As Ridout & Campbell (48) suggested, social networks could be used to test whether there is a change in the communication patterns of users depending on their participation in interventions to address schizophrenia, through social networks. In other cases, we even found the detection of possible suicidal behavior in users diagnosed with schizophrenia (57) (**Table 1**).

It is observed that social networks that use writing as the primary means of communication remain the most important in developing pattern detection algorithms to identify potential schizophrenia patients.

When we analyzed the trivialization and stigmatization continue to be a key element when talking about schizophrenia on social networks since, according to the data (**Table 1**). Furthermore, 15 documents (37.5%) analyze these concepts with the aim of making them visible and educating the population.

It was observed that the social network where this type of behavior by users occurred most frequently was Twitter, which accounted for 80% of the documents (n=12), followed by Youtube with 6.66% (n=1) as the next most frequently used social network to disseminate stigmatizing information about schizophrenia. Finally, it was found that both Weibo (n=1) and Instagram with 6.67% each (n=1).

When analyzing articles focusing on Twitter, studies were found where machine learning was used to detect Tweets containing inappropriate information about schizophrenia that induced confusion and even stigmatization of people with schizophrenia (28, 38, 39).

Furthermore, it was observed that the most frequent method of categorizing stigmatization, and schizophrenia and its diagnosis, was a categorization done by manuscript’s authors (26, 35, 37, 38, 41, 45, 47, 49). Finally, the assessment of stigmatization was provided by health professionals, who participate in social networks and talk about their experiences in finding information about schizophrenia (44).

The analysis of Youtube (53) and Instagram (21), indicates that comments on videos uploaded about schizophrenia were analyzed. Furthermore, on the social network Weibo, an algorithm was developed that allowed the detection of messages that included pejorative words or that diminished the importance of schizophrenia in posts on that social network (40).

Finally, we found 2 articles that focused on analyzing the role of addictions and their relationship with schizophrenia, specifically both articles pertain to writing-based social networks. Fekih-Romdhane, F., et al. (32) assess, through Facebook, the relationship of people with schizophrenia with the risk of excessive use of technology and social networks and possible addictive behaviors. On the other hand, Hswen, Y., et al. (33) examined the possibility that Twitter users who define themselves as people with schizophrenia were more likely to write and post about smoking and to indicate that they used tobacco.

### 3.5 Social networks and categorization of articles schizophrenia conversation on social networks

It is important to emphasize that many of these manuscripts were focused on pattern identification on text-based social networks. Twitter and Weibo appear as analyzed social networks in a total of 435 occasions in the documents found, compared to the use of more audiovisual social networks, such as Instagram, Youtube or TikTok, which appear only in 14 documents.

With respect to text-based social networks, the most used is Twitter, in 25 documents, followed by Facebook with 16. Both are widely used for pattern prediction due to the ease of training models for text recognition, approach observed in 7 documents dealing with Facebook (43.75% of the analyzed documents), and Twitter with a total of 5 documents (20% of the total number of documents dealing with Twitter) (**Table 2**). Finally, in both types of networks, Twitter and Facebook are the only ones that address the topic of schizophrenia and addictions (**Table 2**).

It is necessary to highlight the role of Twitter as a social network where many documents focused on the analysis of the stigmatization of schizophrenia are generated (n=11), following by Prediction of patterns with 8 documents (32% of documents that analyze Twitter). Finally, Mental Health Literacy is discussed in 5 documents (20%) that analyze Twitter.

With respect to audiovisual social networks, it is interesting to note that they appear in only 14 documents, of which YouTube is the most frequently addressed with 10 documents, followed by Instagram with 4. It is striking that there is no document that addresses the analysis of TikTok (**Table 2**). The most addressed topic on YouTube is Mental Health Literacy, with 5 documents (50% of the papers addressing YouTube). Pattern analysis for the prediction of behaviors associated with schizophrenia, is observed in both YouTube (n=3; 30%), and Instagram (n=2; 50%). With respect to Stigmatization of schizophrenia, it is YouTube (n=2; 20%) the network that is most focused on this action (**Table 2**).

When comparing the possible effect of the lockdown on the publication of the number of articles, as well as their subject matter, it is observed that 24 articles (53.33%) were published before the lockdown compared to 21 published afterwards (46.67%).

Comparison of the different categories published between the two periods (**Table 2**) shows that there is no significant difference between the two periods (X^2^ = 2.69; p=0.442).

## 4 Discussion

The purpose of this manuscript is to provide an overview of the current literature on social networks and schizophrenia. To this end, we have organized the following section according to the most frequently mentioned categories in the previous review.

### 4.1 Influence of lockdown

It was observed that, although the total number of documents increased in the period after the lockdown, this increase was not significant in terms of the subject matter addressed.

Although there may have been an increase in the study of topics associated with stigmatization (12) or mental health education (11, 12), there was no significant increase between the two periods. It can be deduced that the interest in analyzing the role of social networks in the approach to schizophrenia remained the same.

### 4.2 Detection of schizophrenia patterns on social networks

The analysis of words, or word combinations, represents an advance in the diagnosis and prediction of possible states of schizophrenia (7–9). Potential therapeutic benefits in its diagnosis have been observed in the social network Twitter. This tool has used a combination of clinical assessment and machine learning, finding significant linguistic differences between people who reported having schizophrenia (14, 30, 38, 43). Healthcare professionals, such as physicians, evaluated the Twitter users’ posts to determine the authenticity of the diagnosis.

In addition, an increased use of first-person pronouns, but also of second-person pronouns, was found in these social network’s texts, which may be indicative of changes in the way individuals think about themselves in relation to others and may be experiencing a worsening of psychotic symptoms (30, 32).

Other studies show that patients with schizophrenia posted images on Instagram with significantly lower saturation, coloration, and number of faces-that is, the images tended to be darker- (14, 61). When assessing the characteristics of the images that were shared on social networks by people with schizophrenia, it was found that: i) when it came to selecting colors, red and black were most frequent, but also ii) in the images in which people appeared, it was found that the faces were blurred (14). There is also a tendency to include differences in the way images are cropped, affecting height and width. These profiles attract fewer followers, possibly due to these differences in image and color characteristics, making their profiles less attractive to other users (14).

It is important to highlight that, beyond changes at the linguistic level, the social networks activity provides digital representations of clinically significant behavioral patterns, potentially associated with psychotic disorders and incipient relapse. Significant increases in tagging behavior and the number of friendships in the month prior to hospitalization for relapse, as well as increased posting activity after midnight and in the early morning, have been identified as signs of inappropriate and disorganized social behavior, often seen when the state of individuals with psychosis worsen (31).

Hswen Y et al (38), linked health behavior such as smoking among social networks users to schizophrenia. Twitter users who identified themselves with schizophrenia spectrum disorder tweeted about smoking more frequently than randomly selected Twitter users from the general population.

Although most of the studies use text-based social networks, it is important to note that audiovisual social networks are those that, to date, have a higher frequency of use (19). Because of this, the need to better understand how these contents are used for the detection of patterns that allow the detection of possible behaviors associated with schizophrenia is essential. In many cases these analyses are based on content analysis of the videos by mental health professionals (65). Likewise, in social networks such as YouTube, it is possible to perform analyses of the interactions of the people who view the videos with respect to them. This allows the introduction of the machine learning process on word analysis (60)

Therefore, analysis of word choice on social networks could help clinicians identify individuals at high risk for schizophrenia before the onset of clinically significant symptoms. Understanding linguistic differences and incremental changes in social networks could create important opportunities for intervention (43, 60). However, although all these data provide clues to possible behavioral changes in patients with schizophrenia, to date it is not clear exactly how social behaviors are manifested through social networks (32, 70).

Newer technologies, such as artificial intelligence and machine learning, will play a central role in future prevention strategies, and finding the right balance between security and ethics will be a major challenge (14, 32, 43). While social networks alone cannot diagnose psychiatric disorders, nor replace the fundamental role of a healthcare professional in psychiatric assessment, they can potentially be used in conjunction with physician information to support clinical decision making (3).

### 4.3 Schizophrenia stigma

The stigmatization of schizophrenia is an issue that we find to be addressed more frequently. This disease is constantly inappropriately mentioned because it is often stigmatized and trivialized (24, 33, 42–44, 46, 49–53) (Not only do patients suffer from psychosis (40), but antipsychotic treatments (42) are also the subject of negative sentiments and judgments by Twitter users (46, 49–53), further demonstrating the persistence of social stigma toward schizophrenia and its treatment (42).

The use of word analysis and machine learning application to detect conversation that use words, adjectives in example, associated to a negative conception of schizophrenia or psychosis. On conversations on social networks (5, 39), analysis methods are also employed to facilitate the automatic detection of stigma toward schizophrenia on social networks (33, 44). The traditional media, as newspapers or television, are a central point for stigmatization of mental health conditions (52), with social networks being a new therapeutic tool to support the disclosure of stigmatized conditions.

According to Li et al (45), 26.22% of relevant Weibo postings showed stigmatizing attitudes towards schizophrenia. Jilka et al (43), also showed that almost half of the public tweets related to schizophrenia in their study were classified as stigmatizing. Alvarez-Mon et al (44), found that terms related to psychosis were associated with a significantly higher number of tweets with negative content, just as people with schizophrenia were more often perceived under negative sentiments, such as unpredictable and dangerous (40–43).

Within these negative tweets, three different stereotypes were identified, suggesting that this disorder hinders the ability to perform some tasks. These are: incoherence, due to the behavioral instability suffered in this disorder; dangerousness, the idea is conveyed that people with the disorder represent a danger to those around them, and relativization, underestimating the suffering of this disorder (43). Regarding the expressions related to the stigma associated with schizophrenia, a greater use of words related to social processes, such as the word “partner,” a more frequent use of the word “kill,” and anger, such as the term “hate”, were noted. Such patterns of language use indicate a higher level of negative sentiment, which may fit into two elements of stigma processes, cognitive dissociation, and negative emotional reactions (40) also describing tweets with negative polarity, such as “Psychiatry breaks people down even more” (43).

These findings confirm the existence of stigma and negative prejudice associated with psychotic disorders. For example, the terms “depression” and “autism” are less likely to be misused than the terms “psychosis” and “psychopath” and “schizophrenia” and “schizophrenic” (43–45). Promoting awareness of the use of words that perpetuate mental health stigma may be beneficial in reducing the stigmatization associated with any mental illness (45, 46).

Education has a positive effect on reducing public stigma towards adults with mental illness. Early identification and intervention in people with psychosis can be achieved through widespread information campaigns and easily accessible services (46, 48).

### 4.4 Mental health literacy

Studies show that there are many users disseminating beneficial information on social networks, increasing public education about schizophrenia and disease management, but also connecting people through organized events focused on this debilitating disorder (35, 39, 41, 48). It is therefore necessary to integrate therapeutic functions using social networks (18) as an aspect of social functioning in psychosis, allowing for the establishment of a more reliable and valid dimension (54, 55).

Responsible use of Twitter by healthcare professionals will increase the visibility of research findings and ensure that up-to-date evidence is easily accessible (41, 48). As the most widely used social network in public health, Twitter can contribute to increasing schizophrenia literacy and academic information in mental health (59, 60). Facebook can be used to track public health awareness of schizophrenia-related issues (35, 41, 54, 70, 71), but too as a tool to know and educate about the pharmacotherapy in elderly, with mental health disorders (68). YouTube can be an educational medium through videos that appeal to younger generations, who are more vulnerable to certain psychiatric disorders (39, 67, 70). These videos may not only help to reduce the length of time that a mental illness goes untreated but may also help to slowly overcome the stigma associated with these disorders (39, 41, 66). However, they will not be suitable for use as educational tools in isolation, as media portrayals of mental illness strongly influence public understanding and may contribute to the harmful stigma associated with psychiatric diagnoses (66).

Therefore, to effectively engage and inform the public on psychiatry-related topics, adequate knowledge based on scientific evidence must be implemented. Healthcare professionals have a responsibility to post content on social networks that fosters mental health awareness (39, 41, 66, 67, 70).

### 4.5 The use of social networks by patients with schizophrenia

Many people with schizophrenia are active users of social networks (18, 48), which may have a positive effect on their subjective well-being, as they develop extensive social networks, communicate frequently with other users, and share personal experiences of living with a mental illness and learning from others (51). Acceptance and usability of the reviewed platforms by patients with schizophrenia were generally high, as were perceptions of usefulness and safety (51, 53, 54).

In analyzing social networks, we see that these technologies promote social connectedness, information seeking and provision, creativity, and recreation. However, there is also the possibility of developing problems related to excessive use. People with mental disorders may be more likely to have psychological risk factors for behavioral addictions (37, 38). These include loneliness, depressive symptoms, or lower life satisfaction, as well as demographic risk factors such as unemployment, low educational attainment, and unmarried marital status. However, the potential benefits of social networks use, such as continued cognitive stimulation and community engagement during periods of increased impairment, may outweigh these risks (18, 54, 55) Therefore, social networks may become a psychoeducational tool for patients with schizophrenia (55, 71).

### 4.6 Limitations and strengths

One of the limitations of this study is that, although we study the influence of social media at the present time, we do not analyze the evolution of the different social networks over time, to gain insight into which ones will be more relevant in the future, and therefore which ones we should pay more attention to as healthcare professionals. An example of this may be the fact that the review was not designed to include from the very beginning the moment when the included social networks were opened to societal participation.

However, a notable strength of the study is that it analyzes the most influential social platforms today, rather than limiting it to just one of them. It also considers multiple diseases, providing a broader view of the current context.

Reviews like this are the first steps that we can take in research to provide a solution to a problem that is becoming increasingly widespread in society.

## 5 Conclusions

Social networks have transformed our daily lives around the world. As technology enables more cohesive and interconnected social networking, better methods of psychoeducational outreach are being developed. Social networks are gaining predictive value in the first episode of psychosis and in episodes of symptom exacerbation, including suicide prevention. They can help ensure that early manifestations of illnesses such as schizophrenia do not go unnoticed, or that severe symptoms in people living with the illness can be identified and addressed with appropriate treatment and support. It is therefore critical to acknowledge that digital platforms can serve as a complement to traditional approaches to disease detection, treatment, and management.

Our findings have shown that mental health stigma is commonplace on social networks. These channels can be used to develop intervention and information strategies aimed at modifying individual and societal health-related behaviors. There are great opportunities to leverage popular social networks platforms to support the mental health and well-being of people with mental illness. By demonstrating success in reaching people who identify themselves with mental illness on social networks, our study also highlights a potentially novel approach to engaging this at-risk group.

This is an emerging area of research. Efforts are clearly needed to determine the feasibility and effectiveness of mental health programs delivered via social networks, and it is also critical to consider risks to privacy, harm, and overall safety. Effective communication by healthcare professionals in disseminating information on social networks is integral to promoting early intervention for psychosis and schizophrenia.

## Author contributions

CS-L: Conceptualization, Data curation, Formal analysis, Writing – original draft. BJ-G: Conceptualization, Formal analysis, Writing – original draft. CR-N: Data curation, Formal analysis, Writing – review & editing. IF-Q: Methodology, Writing – original draft. ER-G: Methodology, Writing – review & editing. CT-D: Visualization, Writing – original draft. IH-P: Conceptualization, Supervision, Writing – review & editing.
